# Co-Activation of Th17 and Antibody Responses Provides Efficient Protection against Mucosal Infection by Group A Streptococcus

**DOI:** 10.1371/journal.pone.0168861

**Published:** 2016-12-28

**Authors:** Xianyang Chen, Ning Li, Shuai Bi, Xiaoguang Wang, Beinan Wang

**Affiliations:** Key Laboratory of Pathogenic Microbiology and Immunology, Institute of Microbiology, Chinese Academy of Sciences, Chaoyang District, Beijing, China; Fundació Institut d’Investigació en Ciències de la Salut Germans Trias i Pujol, Universitat Autònoma de Barcelona, SPAIN

## Abstract

Conserved protein antigens among serotypes of group A *Streptococcus pyogenes* (GAS) have been focused for vaccine development because of the diversity of GAS serotypes and risks of autoimmunity post-GAS infection. Precise delineation of protective immune response to each of GAS antigens is critical for vaccine efficacy and safety. We recently reported that immunization with SrtA of GAS provides Th17-dependent clearance of heterologous serotypes of GAS in NALT. SCPA is a surface virulence molecule of GAS and known to induce antibody-mediated protection against GAS. We hypothesized that co-immunization with SrtA and SCPA would provide more efficient protection by eliciting combined Th17 and antibody responses. The present study showed that mice that were intranasally co-immunized with SrtA/SCPA cleared GAS more efficiently than the mice that were immunized with either SrtA or SCPA individually, and as efficient as the mice that experienced repeated GAS infections. The co-immunization induced Th17 and robust SCPA antibody responses, accompanied by a rapid influx of neutrophils and high myeloperoxidase activity in NALT, suggesting that simultaneous induction of mucosal Th17 and neutralizing antibody responses offers more effective GAS elimination through rapid infiltration and activation of neutrophils. Moreover, Th17 response was strongly induced in mice that experienced repeated GAS-infection and maintained at a high level even after the bacteria were cleared; whereas, it was moderately induced and promptly returned to baseline following bacterial elimination in SrtA/SCPA co-immunized mice. Additional results showed that the survival rate of systemic challenge was significantly higher in infection experienced than in co-immunized mice, indicating that more immune elements are required for protection against systemic than mucosal GAS infection.

## Introduction

*Streptococcus pyogenes*, also known as group A streptococcus (GAS) causes various diseases, ranging from uncomplicated sore throats and invasive, life-threatening infections to immune complications. It remains a major public health burden in both developing and developed countries [1]. Although many efforts have been made, a safe and effective human GAS vaccine is not yet available. The major obstacles that hinder the development of a GAS vaccine are serotype diversity and the potential of autoimmunity related to this pathogen [2, 3].

GAS is known to persist in the pharyngeal mucosa and tonsils, which are the primary reservoirs that are responsible for the maintenance and transmission of GAS to a new host. Murine nasal-associated lymphoid tissue (NALT) is a human tonsil homologue [4, 5]. Like tonsils, NALT plays an important role in antigen uptake for initiation of mucosal immunity [4, 5] and has been used to study mucosal immunity to GAS [6, 7]. Th17 cells and secretory antibodies are critical immune elements against mucosal infections and can be induced in NALT. We previously demonstrated that Th17 response is induced in NALT by intranasal (i.n.) GAS infections [8] or immunization with SrtA [9], a conserved protein that locates inside of the cell walls of GAS, and provides cross protection against various serotypes of GAS [9]. We have demonstrated that SrtA-induced Th17 but not antibody responses play a role in GAS clearance [9] because of the internal location of this protein [10]. These results indicate that Th17 responses contribute significantly to GAS immunity.

Complement component C5a is important for rapid neutrophil recruitment to the sites of streptococcal infection [11] and is required for neutrophil-mediated bacterial killing [12]. C5a peptidase (SCPA), a surface virulence factor, is highly conserved among GAS serotypes. Antibodies directed to SCPA offer protection against multiple serotypes of GAS in mouse experiments [13] and have also been detected in humans [14] but T cell response to SCPA is rarely studied. Recently it is reported that Th17 responses to SCPA is dependent on adjuvants and routes of immunization and is not induced by i.n. immunization [15]. Therefore, it is likely that SCPA provides protection by antibody responses in natural GAS infections. Here we report that SrtA/SCPA co-immunized mice cleared GAS in NALT as efficiently as GAS infection experienced mice, suggesting that SrtA/SCPA may induce mucosal immunity comparable to that induced by nature GAS infection. In addition, more GAS infection experienced mice were survived than SrtA/SCPA co-immunized mice, suggesting that adding more GAS antigens targeting on systemic killing and preventing dissemination to SrtA/SCPA formula may increase efficacy of systemic protection against GAS.

## Materials and Methods

### Ethics statement

This study was performed in strict accordance with the recommendations in the Guide for the Care and Use of Laboratory Animals of the IMCAS (Institute of Microbiology, Chinese Academy of Sciences) Ethics Committee. The protocol was approved by the Committee on the Ethics of Animal Experiments of IMCAS (Permit Number: PZIMCAS2011002). Mice were bred under specific pathogen-free conditions in a laboratory animal facility at IMCAS. All of the animal experiments were conducted under isoflurane anesthesia, and all efforts were made to minimize suffering.

### Bacterial strains and culture conditions

GAS M1 strain (90–226), its isogenic strain containing a spectinomycin-resistant gene (Spec^r^), and M49 strain were obtained from the University of Minnesota. The strains were maintained on sheep blood agar and grown in THB-Neo broth (with 100ug/ml spectinomycin for Spec^r^ strain). Overnight cultures (OD_560_ ~1.1) were washed with and resuspended in PBS and then used for immunization or challenge. CFUs were verified by plating on blood agar plates with or without spectinomycin (100ug/ml) as required [9].

### Cloning and expression of recombinant SrtA and SCPA

Recombinant SrtA protein was cloned and expressed as previously described [9]. Recombinant SCPA, which was comprised of a truncated form of the C5a peptidase that included the replacement of two catalytic residues, was constructed as previously described [7] with slight modification. The recombinant protein lacked a signal sequence, propeptide, and cell wall-spanning and peptidoglycan anchor domains. cDNA encoding the SCPA protein was amplified by PCR from the GAS M1 strain using the following primers: forward 5’-ATGACCATGGCGAATACTGTGACAGAAGACACTCCTGC-3’, reverse 5’-ATGACTCGAGTTGTTCTGGTTTATTAGAGTGGCC-3’. Aspartic acid (D130) and serine (S512) residues were replaced with alanines. The protein was expressed in *Escherichia coli* BL21 (DE3) and purified by affinity chromatography on NiOS2-charged chelating Sepharose Fast Flow gel following repurification by Superdex 200 size exclusion chromatography using an AKTA purifier 2000 system (GE Healthcare Bio-Science AB).

### Mice and intranasal (i.n.) immunization and challenge

Female BALB/c mice (aged 6 to 8 weeks) were purchased from Vital River Laboratory Animal Technology, whose colonies were all introduced from Charles River Laboratories. Mice were anesthetized as previously described [9] and then i.n. inoculated with the indicated antigens in 10 μl PBS (5 μl per nostril). SrtA (10 μg) and SCPA (20 μg) were either used together or separately, combined with cholera toxin B subunit (CTB) (1 μg) (Sigma, St Louis, MO). The dosage of live GAS (M1, M1 Spec^r^, and M49) used for infection was 5 x 10^7^/mouse. Control mice were administered PBS. Mice were immunized three times at 1-week intervals and challenged with GAS M1 (Spec^r^) or M49 strain at a sublethal dose of (2 x 10^8^/mouse) 10–14 days after the last immunization. CFUs in NALT were determined as previously described [9] at 24 hr post challenge.

### Saliva and serum sample collection and antibody measurement by ELISA

Blood was collected via cardiac puncture, and saliva samples were collected by rinsing the oral cavity of each mouse with 0.3 ml PBS. Blood was allowed to clot at room temperature, sera were collected by centrifugation. All of the samples were stored at -20°C. ELISA was conducted as described previously [9] with slight modification. Purified SCPA protein (5μg/well) was bound to flat-bottom microtiter wells (Nalgene Nunc International) in 0.05 M sodium carbonate buffer (pH 9.6) overnight at 4°C. Samples of sera and mouth washes were assayed using two-fold dilutions of either a 1:200 or 1:20 starting dilution. The diluted samples were added to the wells and incubated for 1 h at 37°C. 3, 3', 5, 5'-tetramethylbenzidine staining (Sigma) was added after incubation with horseradish peroxidase-conjugated goat anti-mouse IgG or IgA (1:5000). Antibody titers were defined as the reciprocal of the highest dilution of samples, which yielded an optical density at 450 nm of more than 3 standard deviations above the mean optical density of control samples.

### Cellular staining and flow cytometry

Cellular staining and flow cytometry analyses for T helper cells and neutrophils were conducted as previously described [9]. Briefly, following stimulation with PMA and ionomycin, NALT cells were treated with Brafeldin A and stained for surface markers with anti-CD4-FITC (GK1.5, BD Biosciences) for T helper cells and with anti- CD11b-FITC (M1/70, Biolegend) and anti- GR-1-APC (RB6-8C5, Biolegend) for neutrophils. For intracellular staining, fixed cells were permeabilized and stained with anti-IL-17A-PE (TC11-18H10, BD Biosciences) for Th17, anti-IFN-γ-APC (XMG1.2, BD Biosciences) for Th1 cells and anti-IL-4-PerCP-eFluo^®^710 (11B11, eBioscience) for Th2 cells. Samples were analyzed on a FACSAriaII flow cytometer (BD Biosciences) using FlowJo software (Tree Star).

### MPO activity

The myeloperoxidase (MPO) assay was performed as described by Jeyaseelan et al [16]. NALTs were weighed and grinded in 1 ml MPO buffer (50 mM K_2_HPO_4_ and 0.5% Hexadecyltrimethylammonium bromide, HTAB) and frozen at -80°C. After thawing and refreezing three times, the suspension was centrifuged at 12,000 x g for 30 min at 4°C, and supernatant was collected. An aliquot of 100 μl of supernatant was dispensed into a 96-well plate and then 100 μl of MPO assay buffer (containing 50 mM K_2_HPO_4_, 2 mM *O*-Dianididine dihydrochloride and 0.0005% H_2_O_2_) was added immediately prior to reading the optical density at 450 nm and again 60 s later. MPO activity was calculated by using the following formula: units of MPO activity in each well = (the change in absorbance [between 0 and 60 s]/time [min]) x 1.13 x 10^−2^. MPO activity (Unit) was divided by the weight of NALT tissue.

### Mouse survival assay

Female BALB/c mice (6–8 wk old) were i.n immunized as before (n = 10) and challenged with lethal doses of GAS M1 (3 x 10^8^/mouse) 14 days after the last immunization. Weight loss and survival of infected mice were observed twice a day over a 15-day period. In addition to mice that were found dead (n = 6), mice with weight loss of 20% of the starting body weight were euthanized and recorded as dead. The outcome of mortality was anticipated and approved by the animal ethics committee. At the end of the experiment the survived mice were euthanized by exposure to carbon dioxide gas in a rising concentration.

### Statistics

Statistical analyses were performed using Kruskal-Wallis test for comparison of three or more groups of sample data, Log-rank test for survival rates, and unpaired t-test for others using GraphPad Prism (Version 6.0 for Windows; GraphPad Software). The data were considered significantly different at *P* ≤ 0.05.

## Results

### Co-immunization with SrtA and SCPA induced more efficient GAS clearance than immunization with SrtA or SCPA individually

We hypothesized that activation of both protective Th17 and antibody responses by SrtA and SCPA could provide more efficient protection against GAS.

**Fig 1:**
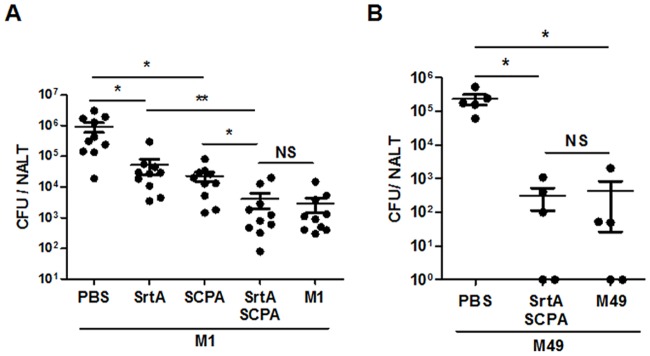
SrtA/SCPA co-immunization induced more efficient protection against GAS infection. (A) Mice were i.n immunized three times at 1-week intervals with indicated antigens (SrtA, 10 μg and SCPA, 20 μg) and CTB or infected with GAS (Materials and Methods). 10 days after the last immunization or infection, mice were challenged with a sublethal dose of GAS M1 (Spec^r^). NALTs were taken and homogenized for CFU counting 24 hr after challenge. Data are geometric means of two independent experiments. (B) Mice were co-immunized with SrtA/SCPA and CTB or infected with GAS M49 (5 x 10^7^) for three times at 1-week intervals and challenged with M49 (2 x 10^8^) 14 days after the last immunization or infection. CFUs in NALT were determined 24 hr after challenge. * *P* ≤ 0.05, **P ≤ 0.01, Kruskal-Wallis test.

Mice were co-immunized i.n. with SrtA and SCPA or with either of them individually. Cholera toxin B was used as an adjuvant. The effect of protection was determined by CFUs recovered from NALT at 24 hr after challenge with the GAS M1 strain. Significantly lower numbers of CFUs were found in SrtA- or SCPA-immunized mice compaired with naïve mice (PBS control). CFU numbers were further reduced in mice that were co-immunized with SrtA and SCPA (Fig 1A), indicating that better protection can be offered by SrtA/SCPA co-immunization. We previously found that mice that experienced repeated intranasal streptococcal infections have robust Th17 response in NALT and clear re-infected GAS in NALT efficiently [8,9]. To compare protective efficacy in co-immunized mice with that in GAS infected mice, CFUs from these mice were determined after challenge. Mice that were immunized with either SrtA or SCPA showed higher numbers of CFUs than those that were recovered from repeated GAS infections; whereas, CFUs in SrtA/SCPA co-immunized mice were reduced as efficiently as in infection experienced mice. These results indicate that more efficient bacterial clearance can be induced by SrtA combined with SCPA than either of them alone and the efficacy is comparable to that induced by live GAS. Because either SrtA or SCPA alone has been shown to provide cross protection to different M types of GAS [9, 13] a broad protection by SrtA/SCPA co-immunization was expected. As shown in Fig 1B, CFUs in SrtA/SCPA co-immunized mice were also efficiently reduced after challenged with serotype M49 strain.

### Th17 response was induced by SrtA but not by SCPA

**Fig 2:**
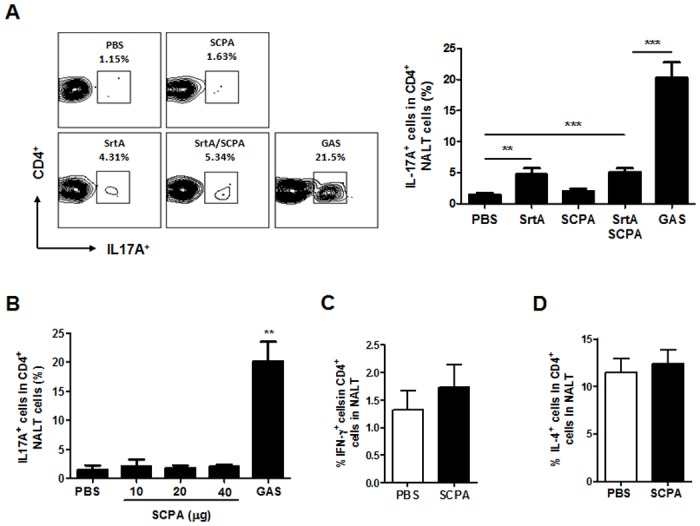
Th17 response was induced by SrtA but not by SCPA. (A) Mice were immunized and challenged as described in Fig 1A. Five days after challenge, mice were euthanized and NALTs were taken. Frequencies of IL-17^+^ cells in CD4^+^ NALT cells were analyzed by flow cytometry and summarized in the bar graph. Data are presented as the means ± SEM (n = 6) from two independent experiments. (B) Mice were i.n immunized with CTB (1 ug) and various dosages of SCPA as indicated or with GAS three times as in Fig 1. NALTs were taken five days after the last immunization or infection and analyzed for IL-17^+^ CD4^+^ cells as in Fig 2A (n = 3). (C and D) Mice were immunized with CTB and 20 μg of SCPA three times and challenged with GAS as in Fig 2A. Five days after the last immunization frequencies of IFN-γ^+^ (C) and IL-4^+^ (D) cells in CD4^+^ NALT cells were determined by flow cytometry. Data are presented as the means ± SEM (n = 7) from two independent experiments. ** *P* ≤ 0.01, Kruskal-Wallis test.

Th17 is an important player in protection against mucosal infection [17] and can be induced by SrtA [9]. To determine whether the co-immunization can induce higher levels of Th17 response Th17 cells in NALT were determined by flow cytometry. Similarly to previously reported [8, 9], Th17 responses in NALT were dramatically induced in infection-experienced mice (>20%) after challenge (Fig 2A), and also significantly increased in mice that had been immunized with SrtA alone or co-immunized with SrtA/SCPA (~ 6%). Interestingly, no Th17 responses were detected in mice that were immunized with SCPA. To confirm this observation and to exclude effects caused by SCPA dosage on Th17 responses, mice were immunized with SCPA at various concentrations and NALT cells were analyzed after challenge. Consistently, no Th17 responses were detected at any of the tested dosages (Fig 2B). Similarly, activation of Th1 and Th2 responses in NALT and in the spleen was not observed in SCPA immunized mice (Fig 2C and 2D). Consistent with these findings, a most recent study shows that i.n. immunization of mice with SCPA does not induce Th17 responses even with Th1/Th17 adjuvant CAF01 [15]. These results indicate that SCPA does not activate T helper cells in the mouse model.

### SrtA/SCPA co-immunization induced high levels of SCPA-specific antibodies

**Fig 3:**
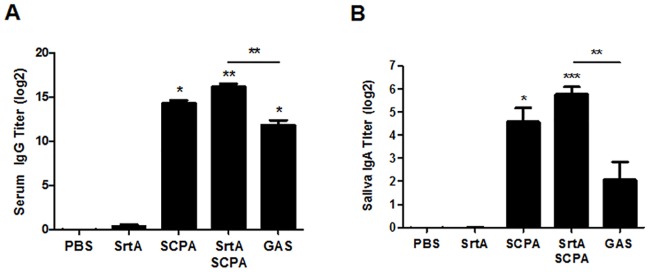
SrtA/SCPA co-immunization induced high levels of SCPA specific antibodies. Mice were immunized and challenged as described in Fig 1A. Serum and saliva samples were taken 24 hr after challenge. (A) Titers of SCPA-specific serum IgG. (B) Titers of SCPA-specific saliva IgA. All of the data are presented as the means ± SEM (n = 10) from at least two independent experiments. * *P* ≤ 0.05, ** *P* ≤ 0.01, *** *P* ≤ 0.001, Kruskal-Wallis test.

It has been shown that administration of anti-SCPA serum via an intranasal route protects mice against streptococcal infections [7], suggesting that the SCPA antibodies in upper respiratory mucosa play critical roles in the prevention of streptococci at the port of entry. Detection of SCPA-specific antibodies by ELISA indicated that both serum IgG and saliva IgA in co-immunized mice were significantly increased and were comparable to those found in mice that were immunized with SCPA alone and even significantly higher than those in GAS infection experienced mice (Fig 3A and 3B). These results indicate that SCPA-specific antibodies were efficiently induced by co-immunization. Because SCPA did not induce T cell responses in this intranasal infection model, the results suggest that the enhanced clearance in SrtA/SCPA co-immunized mice is primarily resulted from SrtA-mediated Th17 activation and SCPA antibodies.

### Neutrophil infiltration and MPO activity were increased in co-immunized mice and correlated with kinetics of GAS clearance

**Fig 4:**
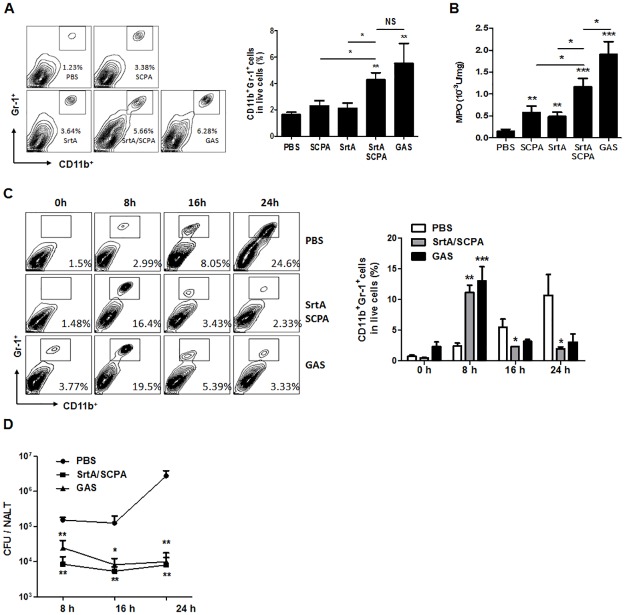
SrtA/SCPA immunization induced rapid neutrophil recruitment to NALT. Mice were immunized and challenged as described in Fig 1A. (A) Four hr after GAS challenge mice were sacrificed. Recruitment of neutrophils to NALT was determined by flow cytometry and summarized in the bar graph. Data are presented as the means ± SEM (n = 12) from at least two independent experiments. (B) Mice were euthanized four hr after challenge, and MPO activity in NALT cells was measured. Data are presented as the means ± SEM (n = 14) from three independent experiments. (C and D) Mice were euthanized at indicated time points, and neutrophil influx (C) was analyzed by flow cytometry on NALT cells and CFUs (D) in NALT were determined on blood agar plates. Data are presented as the means ± SEM (n = 6) from two independent experiments. * *P* ≤ 0.05, ** *P* ≤ 0.01, *** *P* ≤ 0.001, Kruskal-Wallis test.

Evading phagocytic eradication by multiple mechanisms is a major virulence feature of GAS [18]. IL-17 is known to promote neutrophil recruitment, activation, and efficient phagocytic killing [19–21] and efficient GAS clearance is associated with rapid neutrophil infiltration promoted by Th17 activation [9]. On the other hand, neutralizing SCPA would reduce SCPA-mediated cleavage of C5a, which is required for neutrophil-mediated bacterial killing. Our previous study demonstrates that neutrophil infiltration to NALT is significantly increased in SrtA immunized mice by 16 hr after i.n challenge with GAS [9]. We predicted that co-immunization with SrtA/SCPA would result in rapider neutrophil infiltration than immunization with SrtA alone. Flow cytometric analyses demonstrated that as early as four hr after GAS challenge when neutrophil numbers in NALT of SrtA or SCPA immunized mice were similar to those in naïve mice, significantly increased numbers of neutrophils were detected in co-immunized mice (Fig 4A). Myeloperoxidase (MPO) is one of the major antimicrobial systems used by neutrophils. It amplifies the oxidative potential of hydrogen peroxide by generating highly reactive species, such as hypochlorous acid and other oxidants [22, 23]. Although MPO activity in NALT was increased in all immunized mice significantly higher MPO activity was detected in co-immunized mice than in SrtA or SCPA immunized mice (Fig 4B). Noticeably, similar levels of neutrophil influx were observed in infection-experienced and co-immunized mice but higher MPO activity appeared to be induced in infection-experienced mice (Fig 4A and 4B). To better understand neutrophil influx dynamics and its effects on GAS clearance, neutrophils and CFUs in NALT were measured in parallel over a time course of 24 hr post challenge. As shown in Fig 4C, neutrophils in naive mice migrated to NALT slowly and reached their highest levels by 24 hr post challenge. In contrast, higher levels of neutrophils were detected by 8 hr and were then decreased by 16 hr in infection-experienced or co-immunized mice (Fig 4C). Accordingly, CFUs in these mice were substantially reduced by 8 hr and remained low at later time points. Whereas, much higher numbers of CFUs were detected in naïve mice, and they increased by 10-fold by 24 hr (Fig 4D). These results indicate that SrtA/SCPA co-immunization enhances neutrophil activation more efficiently and suggest that more efficient GAS clearance in co-immunized mice is associated with increased neutrophil activation.

### Th17 response in SrtA/SCPA co-immunized mice was resolved promptly following bacterial clearance

**Fig 5:**
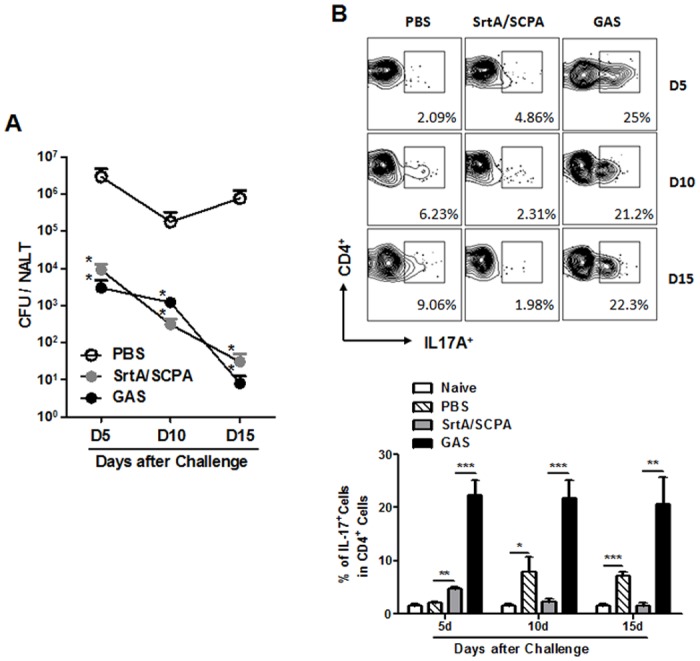
Th17 responses in SrtA/SCPA immunized mice were resolved promptly following bacterial clearance. Mice were immunized and challenged as described in Fig 1A and sacrificed on the indicated days after challenge. (A) CFUs in NALT were determined on blood agar plate. Data are means ± SEM (n = 4). (B) Frequencies of IL-17^+^ cells in CD4^+^ NALT cells were determined by flow cytometry. Data are presented as the means ± SEM (n = 6) from two independent experiments. * *P* ≤ 0.05, ** *P* ≤ 0.01, *** *P* ≤ 0.001, Unpaired t-test (A) and Kruskal-Wallis test (B).

Evidence implicates that GAS-specific Th17 cells are involved in GAS related autoimmune disorders [24]. It is reported that intranasal GAS infection promotes infiltration of GAS-specific Th17 cells into the brain and causes immunopathology in the brain [25]. Therefore, resolution of Th17 responses after elimination of GAS might be important for the safety of GAS vaccines. Mice were co-immunized with SrtA/SCPA or infected with low dose of GAS M1 for three time as before and challenged with spectinomycin-resistant GAS M1 (Spec^r^). CFUs and Th17 cells in NALT were examined over a period of 15 days after challenge. Compared with naïve mice CFUs in co-immunized and infection-experienced mice were significantly reduced on day 5 and further cleared thereafter with similar kinetics (Fig 5A). Th17 cells were dramatically induced in infection-experienced mice and retained at high levels until at least day 15 (Fig 5B, solid black column) when bacteria were cleared (Fig 5A, black dots). However, Th17 cells in SrtA/SCPA co-immunized mice were moderately but significantly increased on day 5 and returned to baseline by day 10 (Fig 5B, grey column), indicating that the moderate levels of Th17 response subside quickly following GAS elimination. These results also suggest that in the presence of SCPA neutralizing antibodies moderate Th17 response could provide efficient mucosal clearance of GAS as well as strong Th17 response does.

### Effects of SrtA/SCPA co-immunization on protection against systemic GAS infection

**Fig 6:**
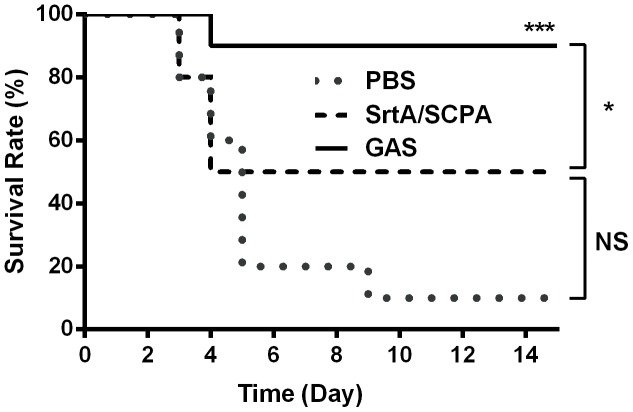
Effects of SrtA/SCPA co-immunization on protection against systemic GAS infection. Mice were grouped (n = 10/group) and immunized three times with SrtA/SCPA or infected with GAS M1 as in Fig 1A. Control mice were administered i.n with PBS. 14 days after the last immunization or infection mice were challenged with a lethal dose of GAS M1 and death rate was recorded daily. * *P* ≤ 0.05, *** *P* < 0.001, Log-rank test.

GAS causes mild and localized mucosal or skin infections and also invasive systemic infections. To determine the role of immune responses induced by SrtA/SCPA on protection against systemic GAS infection, co-immunized and infection experienced mice were challenged i.n. with a high dose of GAS strain M1 (3 x10^8^), which causes bacteremia [26]. Survival of mice was monitored daily over a 15-day period. Death started on day 3 after challenge in naive mice but on day 5 in co-immunized or infection experienced mice (Fig 6). Co-immunization with SrtA/SCPA increased survival from 10% in naive mice to 50% although it was not statistically significance (*p* = 0.5099). Whereas infection experienced mice further increased survival from 50% in co-immunized mice to 90% (*P* = 0.05). These results indicate that SrtA/SCPA co-immunization provides efficient mucosal protection but more immune factors are required for systemic protection when bacteria get into the blood.

## Discussion

Secretory IgA (SIgA) and Th17 are the major elements of mucosal adaptive immunity. SCPA, an important virulence, facilitates the establishment of local infection by GAS and is known to induce antibody-based protection. We previously demonstrate that SrtA of GAS induces Th17 response, which promotes bacterial clearance from NALT independent antibodies. The results in the present study suggest that increased GAS clearance efficiency by SrtA/SCPA co-immunization might be due to eliciting combined Th17 and antibody responses [27].

Neutrophils represent a critical element of innate immunity and are also important effector cells in adaptive immune responses. They are the first type of cell that arrives at sites of infections, wherein they directly attack pathogens. IL-17, produced by Th17 cells, stimulates production of other cytokines and chemokines [20], leading to activation of neutrophils. On the other hand, cell wall-associated streptococcal SCPA protects *S*. *pyogenes* from phagocytosis and interrupt host defenses by enzymatically cleaving C5a [28], [29]. SCPA antibody is able to provide protection by neutralizing SCPA enzymatic activity [7]. Studies have demonstrated that immunization with SCPA delays the accumulation of neutrophils at the foci of streptococcal infection [30] and SCPA^-^ streptococci were cleared more efficiently than wild-type bacteria [31]. Therefore, it is likely that the enhanced GAS clearance in SrtA/SCPA immunized mice could be due to rapider neutrophil infiltration and more efficient killing mediated by SrtA-induced Th17 activation and SCPA-induced antibody production. Further studies are required to demonstrate it conclusively.

We found that Th17 responses were much lower and resolved more rapidly after bacteria were cleared in SrtA/SCPA immunized than in GAS infected mice. Because Th17 cells are also involved in inflammatory and autoimmune diseases moderate levels of Th17 activation by SrtA/SCPA might be beneficial for reduction of Th17-mediated tissue damage. Immunization with SrtA/SCPA intranasally stimulated both mucosal and circulating antibodies significantly, suggesting that the neutralization of SCPA could occur in mucosal and systemic sites. Although immunity provided by SrtA/SCPA was not effective enough to prevent death from systemic infection it did provide significant protection against mucosal infection as effectively as whole GAS cells. Its safety and efficacy make it helpful to prevent GAS infection at port of entry and reduce chances of systemic bacterial spreading from a primary infection. Efficient systemic protection in GAS infection experienced mice suggests that formulating SrtA/SCPA by adding more conserved streptococcal components, such as detoxified IL-8 protease (SpyCEP) and streptolysin O toxins [32], may increase efficacy of systemic protection against GAS.

In summary, the study suggests that induction of both Th17 and antibody responses may provide more efficient protection against GAS infection and that the desired cellular and humoral immune responses could be obtained by selected GAS antigens with defined immunogenicity, such as SrtA and SCPA. Further demonstration of efficient protection elicited by combined Th17 and antibody responses will provide a basis for the development of GAS vaccines with improved safety, efficacy and coverage.
